# Improved outcomes in Familial Adenomatous polyposis management. Results achieved in a single center over a 40-year period

**DOI:** 10.1016/j.clinsp.2025.100717

**Published:** 2025-08-20

**Authors:** Fábio Guilherme Campos, Carlos Augusto Real Martinez, Carlos Frederico Sparapan Marques, Ulysses Ribeiro Jr, Paulo Herman

**Affiliations:** a Colorectal Surgery Division, Gastroenterology Department, Hospital das Clínicas, Faculdade de Medicina da Universidade de São Paulo (FMUSP), São Paulo, SP, Brazil; b Universidade Estadual de Campinas (UNICAMP), Campinas, SP, Brazil; c Universidade São Francisco (USF), Bragança Paulista, SP, Brazil; d Digestive Surgery Discipline, Gastric and Small Bowel Division, Gastroenterology Department, Hospital das Clínicas, Faculdade de Medicina da Universidade de São Paulo (FMUSP), São Paulo, SP, Brazil; e Digestive Surgery Discipline, Hepatic Surgery Division, Gastroenterology Department, Hospital das Clínicas, Faculdade de Medicina da Universidade de São Paulo (FMUSP), São Paulo, SP, Brazil

**Keywords:** Familial adenomatous polyposis, Hereditary colorectal cancer, Proctocolectomy, Prophylactic colectomy

## Abstract

**Introduction:**

Long-term Familial Adenomatous Polyposis (FAP) outcomes depend on timely diagnosis and treatment.

**Purpose:**

To analyse the experience in FAP management over a 40-year period.

**Material and methods:**

Charts from FAP patients (1982‒2023 were reviewed. Demographic data and surgical outcomes were compared between an early (1982 to 2006) and a recent period (2007 to 2023).

**Results:**

176 FAP patients were identified. Mean age at treatment (35.6 vs. 29.7), at diagnosis (35.6 vs. 29.7), and younger than 30-years (30.6 % vs. 50 %) showed that most patients were diagnosed earlier in the recent period. The authors detected a reduction of asymptomatic patients (93.5 % vs. 59.5 %, *p* = 0.0001) and symptoms duration (21.5 vs. 10.6 months, *p* = 0.0001). Simultaneously, both polyposis diagnoses were motivated by family clustering (26.1 % to 57.1 %, *p* = 0.02) and not associated with CRC (40.2 % to 67.8 %, *p* = 0.003) increased. Global CRC incidence decreased from 59.7 % to 32.1 % (*p* = 0.003). Gradually, pouch surgery (69 %) and the laparoscopic approach (92.7 %) turned out to be the most frequent surgical choices. Long-term mortality also diminished (19.5 % to 9.5 %) mainly due to fewer deaths resulting from CRC (11.9 % to 4.8 %). Diagnosis of desmoid disease (9.8 % to 22.6 %) and duodenal cancer (8.7 % to 2.4 %) changed differently.

**Conclusions:**

Comparison of outcomes during a 40-year period revealed a clear improvement in FAP management. This scenario resulted from 1) Continuous orientation to family members to advise an earlier diagnosis; 2) Prophylactic surgical treatment with reduced CRC association, and 3) Increased use of laparoscopic techniques with less morbidity and better outcomes.

## Highlights

• Adenomatous polyposis must be diagnosed and treated before colorectal cancer develops.
• Screening and prophylactic surgery may improve quality of life and survival.
• A review of a 40-year experience demonstrated excellent results and improvement in FAP management.

## Introduction

Around 20 %‒30 % of Colorectal Cancer (CRC) patients may commonly arise as a familial non-syndromic carcinoma, without specific genetic markers, and a second group of 5 %‒6 % will result from well-defined Hereditary Cancer Syndromes, exhibiting a high penetrance of known genetic mutations. These conditions are mainly represented by Lynch Syndrome and Familial Adenomatous Polyposis (FAP) .^1^^,^^2^

Epidemiological reports suggest that FAP represents <1 % of CRC burden worldwide, but it is associated with a great lifetime risk of CRC.^3^ The disease is the most recognizable of the genetic disorders, thanks to its phenotype characterized by the development of a great number of colorectal polyps.^4^

Classical FAP and Attenuated Forms (AFAP) are caused by germline mutations in the APC gene, depending on the codon position of pathogenic variants. Patients usually inherited a defect from paternal origin in an autosomal dominant fashion, although “*de novo*” mutations may occur in about 25 %‒30 %, which explains patients with no family history. The less aggressive variant AFAP exhibits fewer polyps (usually 10‒100), a later mean age of adenoma (44-years) and carcinoma (56-years) appearance, and a 70 % lifetime cancer risk.^5^

Alternatively, a subset of patients will develop multiple colorectal adenomas due to biallelic mutations in the gene MutY Homologue (MUTYH) located on chromosome 1, resulting in an autosomal recessive syndrome called MUTYH-Associated Polyposis (MAP), with a well-documented high lifetime risk of CRC in 43 %‒100 % of cases.^6^

In its early stages and attenuated presentation, FAP is usually asymptomatic. Progressively, symptoms such as bleeding, diarrhea, abdominal pain, and others may appear during the second decade of life. If the disease is not diagnosed and treated, the CRC risk increases with time.^7^ This risk is reduced by prophylactic colectomy followed by surveillance of other primary and secondary cancers (duodenum, pancreas, thyroid, brain, and others) during the evolution of the disease.^8^ Additionally, all first-degree relatives should be properly investigated and tested (when a mutation is confirmed).

Ideally, affected patients and at-risk family members should be included in a registry screening program. In a meta-analysis including 33 studies, it was demonstrated a significant reduction in CRC incidence (by 79 %) and mortality (by 59 %) was demonstrated in families referred to genetic registries and counseling.^9^ Certainly, access to screening programs may improve survival by enabling FAP patients to undergo prophylactic surgery before development of cancer.

In 2006 the authors published an original academic thesis about the experience with FAP management developed in the University Hospital das Clínicas in São Paulo.^10^ Right after that, the authors created and organized a specialized ambulatory with the aim to provide diagnosis, investigation, and follow-up of patients with Hereditary Colorectal Syndromes (mainly Familial Adenomatous Polyposis [FAP]), Lynch Syndrome and Hamartomatous Polyposis Syndromes.

Since then, the authors have personally followed FAP patients treated in the Gastroenterology Department and also those referred from other surgical centers in Brazil. In this setting, the present study has the purpose of evaluating whether the authors have improved the clinical practice regarding the diagnosis and therapeutic management of these patients throughout these years.

## Material and methods

The present investigation was evaluated and approved by the Ethics Committee of the Gastroenterology Department in Hospital das Clínicas (University of São Paulo Medical School, Brazil). Informed consent was not necessary because the focus was only on retrospective data.

All patients admitted to the hospital with FAP diagnosis were identified through the database from the colorectal unit in Gastroenterology Department. Charts from ‘patients considered for surgical treatment from 1982 to 2023 were reviewed to retrieve demographic and surgical data prospectively collected.

With the aim of checking if FAP management improved along the years, the authors verified the following information: age at diagnosis and surgical treatment, sex, race, motive of diagnosis (symptoms of family history), FAP association with CRC, length of symptoms. According to the motive that induced patients to be investigated, patients were categorized as probands when they had no hereditary bowel disease in the family and colonoscopy was indicated on the basis of bowel symptoms. On the other hand, call-up patients were defined as those diagnosed in screening programs or due to the presence of another FAP case in the family.

Prophylactic colectomy was performed at the Colorectal Unit, and all procedures were performed by Brazilian Board-certified colorectal surgeons. Surgical approach (open or laparoscopic) was dependent on the surgeons’ decision according to personal experience and the patient’s features.

Regarding surgical treatment, data included all index abdominal procedures (total colectomy with ileorectal anastomosis – IRA, restorative proctocolectomy with or without mucosectomy and ileal pouch-anal anastomosis – IPAA and total proctocolectomy with ileostomy – TPCI), surgical approach (open or laparoscopic), early day complications and reoperations. During the follow-up period, the authors retrieved diagnoses of desmoid tumors, duodenal neoplasia, mortality rates and causes of death.

The authors assigned the patients to an initial early period of 24-years (from 1982 to 2006) and to a recent period of 16-years duration (from 2007 to 2023), when we started activities in a specialized ambulatory. Then, the authors analyzed whether outcomes have changed by comparing results from these two consecutive periods.

For statistical analysis, Student’s *t*-test or Kruskal-Wallis tests were used for continuous measures (such as age). For other variables (sex, color) there were used Pearson’s Chi-Square or Fisher’s tests. Significance was attested for *p* < 0.05.

The following flow-chart illustrates the numbers of patients included in the present study.

## Results

During the study period, the authors identified 176 FAP patients admitted for surgical treatment. Patient’s demographics are presented in Table 1. According to the proposal for evaluating a supposed temporal progress, they were separated into two groups: 92 patients treated in the early period (1982‒2006) and 84 in the recent period (2007‒2023).

**Table 1:** Demographics of 176 FAP patients admitted for treatment in two periods from 1982 to 2023.

**Variable**		**Early (1982‒2006)**	**Recent (2007‒2023)**	**p-value**
		***n*=92**	***n*=84**	
**Sex**	**n ( %)**	**n ( %)**	**n ( %)**	
Male	72 (40.9 %)	42 (45.6 %)	30 (35.7 %)	0.219
Female	104 (59.1 %)	50 (54.3 %)	54 (64.2 %)	0.219
**Age at treatment (years)**		**M (range)**	**M (range)**	
Average (range)	34.1 (11‒82)	35.6 (17‒82)	29.7 (11‒75)	0.10^a^
Younger than 30y	83 (47.1 %)	39 (42.4 %)	44 (52.4 %)	0.226
**Age at diagnosis (years)**				
Average (range)	31.9 (11‒90)	33.6 (15‒80)	30.1 (11‒63)	0.07^a^
Younger than 30y	97 (55.1 %)	46 (50.0 %)	51 (60.7 %)	0.173
**Color**				
Caucasian		76 (82.6 %)	63 (75 %)	0.267
Non-caucasian		16 (17.4 %)	21 (25 %)	0.267

FAP, Familial Adenomatous Polyposis; M/F, Male/Female; y, Year.

a Fisher Exact Test and Kruskal-Wallis.

There were no significant changes regarding sex distribution or patients’ color. Results concerning mean age at treatment (35.6 vs. 29.7 years, *p* = 0.1), age at diagnosis (35.6 vs. 29.7 years, *p* = 0.07) and percentage of those younger than 30-years (42.4 % vs. 52.4 %; *p* = 0.22) numerically suggest that in the recent period most patients were diagnosed earlier in life when compared to the earlier period, but there was not statistical difference.

In Table 2, the authors present clinical data comparing patients from the two periods. Family history of polyposis was reported by 128 patients (72.7 %), and this number increased (67.3 % to 78.5 %, *p* = 0.12) during the last period. However, when the authors checked what motivated endoscopic evaluation, a small fraction of patients 72 (40.9 %) reported that the reason was family clustering. These called-up patients increased overtime (from 26.1 % to 57.1 %; *p* = 0.02), while probands investigated for symptoms 104 (59.1 %) significantly decreased (73.9 % to 42.8 %, *p* = 0.0001).

**Table 2:** Clinical data of FAP patients treated in two consecutive periods (before or after 2007).

**Variable**		**Early (*n*=92)**	**Recent (*n*=84)**	**p-value**
**FAP family history**				
Yes	48 (27.3 %)	62 (67.3 %)	66 (78.5 %)	0.127
No	128 (72.7 %)	30 (32.6 %)	18 (21.4 %)	0.127
**Motivation for diagnosis**				
Family clustering	72 (40.9 %)	24 (26.1 %)	48 (57.1 %)	**0.02**
Symptoms	104 (59.1 %)	68 (73.9 %)	36 (42.8 %)	**0.0001**
**CRC association**				
Yes	82 (46.5 %)	55 (59.8 %)	27 (32.1 %)	**0.003**
No	94 (53.4 %)	37 (40.2 %)	57 (67.8 %)	**0.003**
**Family history and CRC+**				
Yes	50/128 (39.0 %)	32/62 (36.9 %)	18/66 (27.3 %)	**0.0065**
No	32/48 (66.6 %)	23/30 (76.6 %)	9/18 (50.0 %)	0.112
**Presence of Symptoms**				
Yes	136 (77.3 %)	86 (93.5 %)	50 (59.5 %)	**0.0001**
No	40 (22.7 %)	6 (6.5 %)	34 (40.5 %)	**0.0001**
**Symptoms duration (months)**				
Average^a^		21.5 (0‒240)	10.6 (0‒48)	0.004^a^
<6-months		32 (34.8 %)	44 (52.4 %)	**0.002**
**Clinical setting**				
Curative	163 (92.6 %)	83 (90.2 %)	80 (95.2 %)	0.255
Palliative	13 (7.4 %)	09 (9.8 %)	04 (4.8 %)	0.255
**Non-colorectal tumors**				
Desmoid	28 (15.9 %)	09 (9.8 %)	19 (22.6 %)	**0.002**
Gastroduodenal	10 (5.7 %)	08 (8.7 %)	02 (2.4 %)	0.100

FAP, Familial Adenomatous Polyposis; CRC, Colorectal Cancer.

a Fisher Exact Test and Kruskal-Wallis.

Within the group with family history, 50 patients were diagnosed with CRC (39.0 %), and this incidence was significantly reduced over the 2 periods (36.9 % to 27.3 %, *p* = 0.006). However, among patients with no family history (32 CRC cases in 48 probands), the reduction in CRC incidence over time was not significant (76.6 % to 50.0 %, *p* = 0.11).

Simultaneously, the was a decrease in symptom presence (93.5 % vs. 59.5 %; *p* = 0.0001) and duration (21.5 to 10.6 months, *p* = 0.0001) before surgery. As a possible consequence, the proportion of associated CRC dropped from 59.8 % to 32.1 % (*p* = 0.0003) in the recent period. A palliative treatment scenario remained stable in both periods (9.8 % and 4.8 %, *p* = 0.25). Finally, while desmoid tumor diagnosis rates were greater (22.6 % × 9.8 %; *p* = 0.02) among patients from the first group, while gastroduodenal cancer diagnosis diminished with no statistical significance (2.4 % × 8.7 %; *p* = 0.10).

Table 3 presents the carcinoma incidence (tumoral mass or malignant polyp) diagnosed in each decade of life. Comparison of two periods showed that a CRC reduction was diagnosed in the second period as age progresses, especially after 40 years.

**Table 3:** Number of colorectal cancers and FAP patients found in each decade of life among 176 patients.

**Total (decade of age)**			**Early phase (92)**		**Recent phase (84)**		**P**
**Decade**	**CRC/FAP**	**%**	**CRC/FAP**	**%**	**CRC/FAP**	**%**	**value**
10 - 20y	10 / 44	22.7 %	05 / 18	27.7 %	05 / 26	19.2 %	0.71
21–30y	22 / 51	43.1 %	16 / 29	55.2 %	06 / 22	27.2 %	0.08
31–40y	20 / 37	54.0 %	12 / 21	57.1 %	08 / 16	50.0 %	0.74
41–50y	14 / 21	66.6 %	10 / 11	90.9 %	04/10	40.0 %	**0.02**
51–60y	10 / 15	66.6 %	07 / 07	100 %	03/08	37.5 %	**0.02**
61–70y	05 / 07	71.4 %	04 / 05	80.0 %	01/02	50.0 %	1.00
71–80y	01 / 01	100.0 %	01 / 01	100 %		0	1.00
**CRC**	**82 / 176**	**46.6 %**	**55 / 92**	**59.7 %**	**27 / 84**	**32.1 %**	**0.003**

CRC, Colorectal Cancer; N, Number of tumors; %, Percentage; y, Years; FAP, Familial Adenomatous Polyposis. Fisher Exact Test.

In Table 4 there are listed all index procedures are listed after exclusion of 6 (3.3 %) patients submitted to segmental resection, intestinal deviation, or even non-operated (due to individual features). Classical resections were performed in 170 patients: restorative proctocolectomy with Ileal Pouch-Anal Anastomosis (IPAA, 49.4 %), total colectomy with Ileo-Rectal Anastomosis (IRA, 38.2 %), and Total Proctocolectomy with Terminal Ileostomy (TPCI, 12.3 %). While the indication of pouch surgery increased (29.8 % to 69.8 %, *p* = 0.0001), TPCI (19.5 % to 4.8 %, *p* = 0.005) and IRA (50.5 % to 25.3 %, *p* = 0.01) received fewer indications over time.

**Table 4:** Classical surgical procedures and abdominal approaches performed in two consecutive periods (early and recent periods) in 170 patients.

**Procedure**	**Total (*N*=170)**		**Early (*N*=87)**		**Recent (*N*=83)**		**P value**
	***N***	***%***	***N***	***%***	***N***	***%***	
IPAA	84	49.4 %	26	29.8 %	58	69.8 %	**0.0001**
IRA	65	38.2 %	44	50.5 %	21	25.3 %	**0.01**
TPCI	21	12.3 %	17	19.5 %	4	4.8 %	**0.005**
**Approach**							
Open	84 / 170	(49.4 %)	78/87	89.6 %	6/83	7.2 %	**0.0001**
Laparoscopic	86 / 170	(50.6 %)	9/87	10.3 %	77/83	92.7 %	**0.0001**

TPC/I, Total Proctocolectomy with end Ileostomy; IPAA, Restorative Proctocolectomy with stapled Pouch-Anastomosis; IRA, Total colectomy with Ileorectal Anastomosis.

Fisher Exact Test.

There was no difference in the type of abdominal approach [84 open (49.4 %) × 87 laparoscopic (50.6 %)]. However, laparoscopic procedures were more indicated in the last period (92.7 % × 10.3 %, *p* = 0.0001).

Complications of each operative procedure were compared, and results are presented in Table 5. Surgical complications were detected in 37 among 170 patients (21.7 %), with no increased rate of complications over the two periods (17.2 % vs. 26.5 %; *p* = 0.19). Reoperations were necessary in 12 cases (7.0 %), with no differences between IPAA and IRA (9.5 % and 6.1 %, respectively *p* = 0.55). No 30-day mortality was registered in the present series.

**Table 5:** Complication rates after surgical resections in 170 FAP patients during early and recent periods.

**Period**	**TPCI (*n*=21)**	**IRA (*n*=65)**	**IPAA (*n*=84)**	**Total ( %) 170**
Early (87)	1/17 (5.9 %)	7/44 (15.9 %)	7/26 (26.9 %)	15 (17.2 %)
Recent (83)	0/4 (0)	6/21 (28.5 %)	16/58 (27.5 %)	22 (26.5 %)
P value	1.00	0.321	1.00	0.19
Total (170)	1 (4.8 %)	13 (20 %)	23 (27.4 %)	37 (21.7 %)

TPCI, Total Proctocolectomy with end Ileostomy; IPAA, Restorative Proctocolectomy with stapled Pouch-Anastomosis; IRA, Total colectomy with Ileorectal Anastomosi; N, Number of patients.

Statistical analysis: Fisher Exact Test.

Table 6 presents death rates registered in 26 patients (14.7 %). The global mortality rate decreased numerically (without clear significance thresholds) from 19.5 % to 9.5 % (*p* = 0.08) in the recent period. There was also a numerical reduction of CRC as a cause of death among all patients (from 11.9 % to 4.8 %; *p* = 0.1) and among all causes of death in the recent period (from 61.1 % to 50 %; *p* = 0.68). Simultaneously, there was a numerical increase of non-colorectal cancers and other causes of death (from 38.9 % to 50 %; *p* = 0.68).

**Table 6:** Causes of death of 26 patients during early and recent phases.

**Causes of death**	**Early (92)**	**Recent (84)**	**p-value**
***n*=26 (14.7 %)**	***n*=18 (19.5 %)**	***n*=8 (9.5 %)**	***p*=0.08**
**Colorectal cancer, *N*=15 (9.4 %)**			
Among all patients	11/92 (11.9 %)	4/84 (4.8 %)	0.10
Among all deaths	11/18 (61.1 %)	4/8 (50.0 %)	0.68
**Other causes, *N*=11 (6.2 %)**			
Of all patients	7/92 (7.6 %)	4/84 (4.8 %)	0.54
Of all deaths	7/18 (38.9 %)	4/8 (50.0 %)	0.68

Statistical analysis: Fisher Exact Test.

## Discussion

Management of FAP patients experienced a remarkable improvement in recent decades, thanks to the crescent knowledge of the disease behavior, to the introduction of new technologies and better selection criteria for surgery.^11^ Identification of individuals at risk and prophylactic surgery may help to reduce CRC incidence, extend life expectancy, improve quality of life, and modify causes of mortality.^8^^,^^12^^,^^13^ With this in mind, the authors analyzed the management of the FAP patients during the last four decades, by comparing outcomes before and after 2007.

As stated by the recent ASCRS Clinical Practice Guidelines, the most important clinical feature of FAP lies on the chances to develop CRC earlier in life.^6^ CRC incidence usually increases with age at diagnosis, being rare before 20-years, and the reason why prophylactic colectomy must be performed around this age.^14^ Important Polyposis Registries from Sweden and Japan reported mean ages of 22 and 28 years in asymptomatic patients, respectively, while the mean age of subjects with CRC was 40 years.^15^^,^^16^ In another recent study in Japan, the records of 147 patients from 23 specialized institutions undergoing prophylactic colectomy revealed a greater incidence (23 % vs. 10 %) of malignant polyps in patients with a greater median age (31 vs. 27 years) .^17^ For sure, this fact influences prognosis. In this respect, the Italian Registry showed that postsurgical survival among patients without CRC at colectomy was 68 % after 30-years, compared to 41 % after 10-years in those with cancer.^18^

The comparison of the two periods in the manuscript demonstrated an important reduction in average age at treatment (35.6 to 29.7 years) and CRC association (from 59.8 % to 32.1 %), especially among patients older than 40-years (from 91 % to 40 %). These figures are quite similar to the ages (27 and 31 years) at the time of prophylactic surgery observed in two consecutive groups (2000‒2006 and 2007‒2012) of the Japanese FAP Registry.^17^ Also, the Danish Polyposis Registry reported a 50 % reduction of cancer rates between 1980 and 2020, in which the CRC risk was a main contributor to the diminished global risk overtime.^8^

Besides age, patient’s phenotype must also guide surgical decisions. In a multi-institutional study, a group of 303 patients revealed CRC in 38 %, and the cut-off age for predicting CRC in attenuated, sparse and profuse phenotypes was 46-, 31- and 27-years, respectively.^19^ These authors suggested different schedules for surgery according to profuse and sparse phenotypes (before their mid-to-late 20-years) or attenuated (individual decision).

In the present series, results in the recent group showed a numerical reduction in mean ages at surgical treatment without statistical significance. Fortunately, patients within this group were also less frequently symptomatic (59.5 % vs. 93.5 %), had a reduced length of symptoms (10.6 vs. 21.5 months), and CRC association at diagnosis (32.1 % vs. 59.8 %). In 2010, the published results of this group revealed a worst scenario, where only 10 % were asymptomatic (versus 40 % today) and association with CRC was found in 60 % (versus 32 % today) at diagnosis. Similar data have been previously reported in Italy, where the age at diagnosis among 604 patients was studied in relation to the presence of symptoms at presentation and the presence of CRC at surgery. The authors reported a 3.3 % risk of colonic cancer in asymptomatic patients younger than 30-years, against 80 % among symptomatic patients older than 40-years.^18^

Confrontation of CRC incidence in each decade of life in the patients reveals that 43 % had malignant tissue already detected in the pathological specimen of patients operated during their third decade of life. Fortunately, the authors achieved a persistent reduction in CRC incidence when comparing the two periods in all age ranges, especially after the fourth decade, in which this diagnosis fell from 59.7 % to 32 %.

The reason why the patient undergoes a diagnostic colonoscopy is another important factor influencing CRC incidence. In the literature, many single-center observational studies and reviews have already demonstrated a greater incidence of CRC in symptomatic patients when compared to those investigated by screening.^20^

According to the presence of other FAP cases in the family, patients were classified as a called-up group, in which the possibility of an earlier diagnosis is theoretically greater, because the information of an affected relative may influence the individual’s decision to be investigated even when there are no clinical complaints. Conversely, if the patient is the first family member (a proband) diagnosed with FAP, he will probably have a colonoscopic examination only if and when symptoms appear. If colorectal polyps are found, the proband should preferably be tested and his relatives screened for a detected mutation or for the presence of polyps. Considering that FAP is already an uncommon diagnosis, the situation of not referring family history (in about 30 % of cases) to guide the search for diagnosis turns to be an unfavorable condition.

In accordance with the literature findings, only 48 (27.3 %) of the 176 patients were considered probands with no family history, unaware of their disease, commonly symptomatic, and more often with associated CRC. From the early to the recent group, representation of probands diagnosed by symptoms diminished (73.9 % to 42.8 %, *p* = 0.0001), what may be considered an excellent outcome, as CRC incidence has fallen from 76.6 % to 50 % over time. Simultaneously, the authors diagnosed 50 CRCs in the called-up group (39 %), which also exhibited a CRC reduction (36.9 % to 27.3 %, *p* = 0.006). Interestingly, the rates of familial clustering leading to colonoscopic investigation increased in over half of all patients (26.1 % to 57.1 %) in the recent period. Probably, this may have occurred because the authors routinely raise this issue with patients and all family members in the daily clinical practice.

The value of screening in CRC incidence was clearly demonstrated in the Danish Registry, showing CRC in 61.6 % of probands versus 1.9 % among call-up patients^21^ similar to reports of many other European Polyposis Registries.^7^

Since colonoscopic surveillance does not guarantee control of a carcinogenic mucosa, surgery apparently seems to be the most effective way to prevent CRC in a long-term scenario.^5^ But surgery must be timed judiciously, chosen wisely, and performed expertly.^22^^,^^23^ In addition to more traditional metrics such as medical co-morbidities and nutritional status, this shared decision must establish a moment that suits age, severity of polyposis, educational, intellectual, and emotional development of the patient.^24^ Certainly, preferences and experience of healthcare providers may influence the final decision, and the risk of developing desmoid tumors must not be minimized in the surgical planning.^25^^,^^26^

The present series comprises 170 classical resections to treat FAP. Fortunately, TPCI has been less frequently required in this experience (12 %), being reserved for rare circumstances such as a dysfunctional sphincter, extensive mesenterial desmoids, locally advanced rectal cancer, cases requiring radiotherapy, or when the patient refused functional sequelae of pouch surgery. On the other hand, TPCI is responsible for a small number of complications (5 % in this series), as it does not involve anastomosis.

In most patients, surgical choice varied between IPAA (47.7 %) and IRA (36.9 %). Both are considered complex operations that may bring important changes in bowel habits even in the absence of complications. Since its introduction, IPAA has been considered the preferred procedure, despite its associated morbidity.^27^ There exists an estimated low risk of cancer in the pouch body (0.01 %)^28^ and in the rectal cuff (1.1 %‒1.9 %).^29^ On the other hand, IRA is usually associated with better functional results, but the overall risk of rectal cancer is greater (6 %‒11 %).^25^

The authors observed surgical complications in 37 (21.7 %) patients, with similar complication and reoperation rates throughout time. Complications may result in multiple surgeries, the need for a stoma, and the threat of desmoid disease. This is the main reason why the authors don’t agree with the idea of 1-stage IPAA for FAP patients, due to associated greater complication rate.^30^

In the recent period, IPAA and the laparoscopic approach indications increased, theoretically affecting in opposite directions the global surgical risks. The laparoscopic access comprised 93 % of the procedures in the recent period. Especially in the young FAP population, laparoscopy signifies a less invasive and more cosmetic option that will probably result in a faster and painless recovery. Thus, the opportunity to perform a laparoscopic procedure in this population has been considered a positive evolution in their management, despite the eventual selection bias in comparative studies showing several advantages over open resections.^11^^,^^31^^,^^32^

CRC, desmoid disease and duodenal tumors are important causes of death in FAP. The global death rate was 14.7 %, with a tendency of reduction (19.5 % to 9.5 %, *p* = 0.08) in the recent period. CRC was responsible for death in 9.4 % of the patients. More importantly, the authors also noticed a reduction in CRC-related death among all patients (11.9 % to 4.8 %) and among all causes of death (61.1 % to 50 %). Compared to the previous publication about this subject in which CRC was implicated in 14 % of deaths,^32^ it seems the authors are in the right direction towards reducing deaths from CRC.

Simultaneously, the authors found a numerical increase participation of other causes of death in the whole series (38.9 % to 50 %). With prophylactic colectomy and CRC surveillance, long-term evolution may show a greater risk of secondary primary tumors.

Within this context, desmoid disease rate (22.6 % × 9.8 %; *p* = 0.002) was greater in the recent group, while gastroduodenal cancer diagnosis diminished (2.4 % × 8.7 %) with no statistical difference (*p* = 0.1).

Although development of desmoids in FAP has been linked with many risk factors (extracolonic manifestations, sex, family history, genotype, pouch surgery procedure),^33^ the preponderance of female patients and the greater number of IPAA in the recent period may explain these results. However, the increased desmoid diagnostic rate may also be justified by a more effective availability of radiological resources associated with a focused attention to this unfortunate condition during the last decades.^31^^,^^34^^,^^35^

Desmoid tumors may affect a reasonable proportion of FAP patients (20 %‒30 %), with a profound impact on their quality of life. For this reason, the authors carefully postpone prophylactic treatment in at-risk patients to avoid surgical trauma. Regarding the observed reduction (8.7 % to 2.4 %) of upper digestive tumors, the authors believe it is probably the consequence of the selective surveillance strategy adoption. Lifetime risk of duodenal adenomas is around 70 % and duodenal cancer is estimated to affect 4 % to 18 %, being recognized as the second-leading cause of mortality in FAP.^25^^,^^36^^,^^37^ Thus, recommendations for upper endoscopic procedures have changed from an observational to an interventional attitude, in which cancer prevention is achieved by removing precursor lesions.^21^^,^^38^

This evolutional variation has historically been observed by many reference centers and national registries in developed countries. For example, data from the Canadian Registry indicate that periampullary tumors became more frequent after the 1970s, when colorectal prophylactic surgery became routine.^39^ In Japan, results from two consecutive series^16^^,^^40^ showed a fall in death age due to a reduction in CRC, with subsequent elevation of death caused by DT (from 0.7 % to 10 %) in a period of thirteen years.

Health care providers involved in FAP management should be constantly alert of new perspectives and ideas. In a recent manuscript comparing patients treated before or after 2000, investigators from Leiden University Medical Center report that today they perform surgery for FAP less often, and surgery has been safely postponed to a more advanced age. They stated that the most important endoscopic indication for surgery is a substantial number of large adenomas of > 5‒10 mm.^14^

The group from the Cleveland Clinic has recently proposed snare polypectomy in 2‒3 sessions or even endoscopic mucosal resection to control rectal polyps, which they called a multistage rectal polypectomy with the aim of avoiding an immediate pouch surgery at least in the short term [41].

Another important issue is the question of how much the risk of desmoid should guide surgical decisions. IPAA has been considered a desmoidogenic operation. Despite the absence of general agreement about this, at-risk patients usually delay surgery and preferably undergo IRA with medical or endoscopic clearance of rectal polyps. Avoidance of IPAA in favor of IRA is also based on the possibility of sexual and reproductive dysfunctions, mainly in those without CRC, obese or very young patients.

It is not always easy to establish a perfect temporal correlation between the establishment of the specialized ambulatory in 2007 and all the favorable outcomes the authors have obtained since then. However, the present manuscript suggests this probable association, as the authors presented data revealing that the efforts really improved long-term results during the last four decades.

In fact, since 2007 FAP patients were diagnosed earlier in life, with a greater proportion of asymptomatic and CRC-free polyposis. In effect, early diagnosis via family screening reduced CRC incidence from 59.8 % to 32.1 % (*p* = 0.003). All these efforts apparently reduced deaths from CRC. Concerning innovative techniques, it was possible to use the laparoscopic approach in most (70 %) of surgical procedures, with no deterioration of operative outcomes directly associated with a different approach. Otherwise, the authors currently use minimally invasive techniques with the belief that they provide substantial advantages to this young population concerning postoperative recovery, preservation of the abdominal wall and body image. Also, the morbidity rates are similar to those reported in the literature, despite the fact that procedures are performed by medical residents who still have to surpass the learning curve associated with laparoscopic surgery.

Moreover, overall mortality declined from 19.5 % to 9.5 % (*p* = 0.08), driven by fewer CRC-related deaths. Regarding causes of mortality, it seems that patient surveillance has allowed not only the most effective diagnosis and treatment of desmoid disease over the years, but it has also influenced the prevention of duodenal cancer by controlling duodenal mucosa over the disease evolution.

The authors must report here some limitations of this present study. Despite being the largest Brazilian cohort (and probably in South America), FAP is a relatively not so common disease, and thus comparison of some small numbers may not reveal statistical differences. Moreover, the authors present here outcomes from a single-center with low-resource features resulting from its public nature, such as a lack of genetic data that could eventually help and influence clinical and surgical decisions throughout time.

Therefore, the present manuscript clearly suggests as effective improvement in the management of FAP patients during this 40-year period in the studied institution. But the authors recognize there is still a long way ahead. Despite the cost of a genetic analysis, integration of genotype data into the practice would probably refine surgical indications and surveillance decisions in some cases. Moreover, the authors need to request a greater participation of social and psychological assistance to deal with at-risk people who refuse screening examinations despite repeated requests. Most importantly, education and counseling about all the disease’s challenges with family members may certainly improve their engagement in screening. Similarly, a close contact with those afraid of operations could also avoid delayed surgical procedures. For the future, the authors may conduct a multicenter study to validate these findings across diverse populations.

## Conclusions

In the studied institution, the evolutionary comparison of FAP patients’ outcomes reveals a clear improvement in their management during the last four decades. This favorable scenario resulted mainly from 1) A continuous orientation to family members to advise an earlier diagnosis; 2) Prophylactic surgical treatment with reduced CRC association; and 3) Good short-term operative outcomes with an increased use of minimally invasive procedures, aiming to benefit this young population.

## Authors' contributions

Campos F contributed to conceptualization, data curation, formal analysis, methodology, writing (re: CFS, Ribeiro Jr. U and Herman P contributed with formal analysis and critical review.

The authors state that the present article has not been published previously and is not under consideration for publication elsewhere. This final version of the manuscript was approved by all authors. This publication is approved by all authors and tacitly or explicitly by the responsible authorities where the work was carried out, and that, if accepted, it will not be published elsewhere in the same form, in English or in any other language, including electronically, without the written consent of the copyright- holder.

The authors ensure the present work has been carried out in accordance with the Ethics Code of the World Medical Association.

On behalf of all authors, I disclose no financial or personal relationships that could inappropriately influence the present work.

## Declaration of competing interest

The authors declare no conflicts of interest.

## References

[1] Jasperson K.W., Tuohy T.M., Neklason D.W., Burt R.W. (2010). Hereditary and familial colon cancer. Gastroenterology.

[2] Lynch H.T., Shaw T.G. (2013). Practical genetics of colorectal cancer. Chin Clin Oncol.

[3] Horisberger K., Mann C., Lang H. (2023). Current surgical concepts in Lynch Syndrome and familial adenomatous polyposis. Visc Med.

[4] Campos F.G., Figueiredo M.N., Martinez C.A.R. (2015). Colorectal cancer risk in hamartomatous polyposis syndromes. World J Gastrointest Surg.

[5] Adla A., Zheng M., Singhal M. (2023). An unsuspecting case of familial adenomatous polyposis (FAP). Cureus.

[6] Poylin V., Shaffer V.O., Felder S.I., Goldstein L.E., Goldberg J.E., Kalady M.F. (2024). The American Society of Colon and Rectal Surgeons Clinical Practice Guidelines for the management of inherited adenomatous polyposis syndromes. Dis Colon Rectum.

[7] Campos F.G., Nicácio De Freitas I., Imperiale A.R., Seid V.E., Perez R.O., Nahas S.C. (2010). Colorectal cancer in familial adenomatous polyposis: are there clinical predictive factors?. Cir Esp.

[8] Karstensen J.G., Bülow S., Højen H., Jelsig A.M., Jespersen N., Andersen K.K. (2023). Cancer in patients with familial adenomatous polyposis: a nationwide Danish cohort study with matched controls. Gastroenterology.

[9] Barrow P., Khan M., Lalloo F., Evans D.G., Hill J. (2013). Systematic review in the impact of registration and screening on colorectal cancer incidence and mortality in familial adenomatous polyposis and Lynch syndrome. Br J Surg.

[10] Campos F.G.C.M. (2006).

[11] Moreira A.L., Church J.M., Burke C.A. (2009). The evolution of prophylactic colorectal surgery for familial adenomatous polyposis. Dis Colon Rectum.

[12] Mallinson E.K.L., Newton K.F., Lalloo F., Clancy T., Hill J., Evans D.G.R. (2010). The impact of screening and genetic registration on mortality and colorectal cancer incidence in familial adenomatous polyposis. Gut.

[13] Komori K., Kanemitsu Y., Kazunori K., Kimura K., Yawata K., Shimizu Y. (2013). Efforts to advance surgical treatments for patients with familial adenomatous polyposis for 40-years in a cancer hospital. Hepatogastroenterology.

[14] Vasen H.F.A., Ghorbanoghli Z., Ruijter, Trinidad Ro (2019). Optimizing the timing of colorectal surgery in patients with familial adenomatous polyposis in clinical practice. Scand J Gastroenterol.

[15] Bjork J., Akerbrant H., Iselius L., Alm T., Hultcrantz R. (1999). Epidemiology of familial adenomatous polyposis in Sweden: changes over time and differences in phenotype between males and females. Scand J Gastroenterol.

[16] Iwama T., Tamura K., Morita T., Hirai T., Hasegawa H., Koizu-Mi K. (2004). Japanese Society for Cancer of the Colon and Rectum. A clinical overview of familial adenomatous polyposis derived from the data-base of the Polyposis Registry of Japan. Int J Clin Oncol.

[17] Yamadera M., Ueno H., Kobayashi H., Konishi T., Ishida F., Yamaguchi T. (2017). Current status of prophylactic surgical treatment for familial adenomatous polyposis in Japan. Surg Today.

[18] Bertario L., Presciuttini S., Sala P., Rossetti C., Pietroiusti M. (1994). Causes of death and postsurgical survival in familial adenomatous polyposis: results from the Italian Registry. Italian Registry of Familial Polyposis Writing Committee. Semin Surg Oncol.

[19] Kobayashi H., Ishida H., Ueno H., Hinoi T., Inoue Y., Ishida F. (2017). Association between the age and the development of colorectal cancer in patients with familial adenomatous polyposis: a multi-institutional study. Surg Today.

[20] Croner R.S., Brueckl W.M., Reingruber B., Hohenberger W., Guenther K. (2005). Age and manifestation related symptoms in familial adenomatous polyposis. BMC Cancer.

[21] Karstensen J.G., Burisch J., Pommergaard H.C., Aalling L., Højen H., Jespersen N. (2019). Colorectal cancer in individuals with familial adenomatous polyposis, based on analysis of the Danish polyposis registry. Clin Gastroenterol Hepatol.

[22] Sommovilla J., Liska D., Church J. (2021). Multistage rectal polypectomy: an alternative to proctectomy in patients with familial adenomatous polyposis. Dis Colon Rectum.

[23] Koskenvuo L., Ryynänen H., Lepistö A. (2020). Timing of prophylactic colectomy in familial adenomatous polyposis. Colorectal Dis.

[24] Campos F.G. (2014). Surgical treatment of familial adenomatous polyposis: dilemmas and current recommendations. World J Gastroenterol.

[25] Aelvoet A.S., Buttitta F., Ricciardiello L., Dekker E. (2022). Management of familial adenomatous polyposis and MUTYH-associated polyposis; new insights. Best Pract Res Clin Gastroenterol.

[26] Campos F.G., Araújo S.E., Melani A.G., Pandini L.C., Nahas S.C. (2011). Cecconello I. Surgical outcomes of laparoscopic colorectal resections for familial adenomatous polyposis. Surg Laparosc Endosc Percutan Tech.

[27] Miller-Ocuin J.L., Dietz D.W. (2022). The evolution of pelvic pouch surgery: optimal pouch design for an ileal pouch anal anastomosis. Clin Colon Rectal Surg.

[28] Boostrom S.Y., Mathis K.L., Pendlimari R., Cima R.R., Larson D.W., Dozois E.J. (2013). Risk of neoplastic change in ileal pouches in familial adenomatous polyposis. J Gastrointest Surg.

[29] Pasquer A., Benech N., Pioche M., Breton A., Rivory J., Vinet O. (2021). Prophylactic colectomy and rectal preservation in FAP: systematic endoscopic follow-up and adenoma destruction changes natural history of polyposis. Endosc Int Open.

[30] Campos F.G. (2013). Current trends regarding protective ileostomy after restorative proctocolectomy. J Coloproctol.

[31] Campos F.G., Martinez C.A., Novaes M., Nahas S.C., Cecconello I. (2015). Desmoid tumors: clinical features and outcome of an unpredictable and challenging manifestation of familial adenomatous polyposis. Fam Cancer.

[32] Campos F.G., Perez R.O., Imperiale A.R., Seid V.E., Nahas S.C., Cecconello I. (2010). Evaluating causes of death in familial adenomatous Polyposis. J Gastrointest Surg.

[33] Yang W., Ding P.R (2023). Update on familial adenomatous polyposis-associated desmoid tumors. Clin Colon Rectal Surg.

[34] Campos F.G., Bustamante-Lopez L., Kotze P.G., Martinez C.A.R. (2021). Variables affecting desmoid tumor risk in Familial Adenomatous Polyposis: a critical review. Cancer Rep Rev.

[35] Campos F.G., Martinez C.A.R., Bustamante-Lopez L.A., Mendonça R.L.S., Kanno D.T. (2023). Intra-abdominal desmoid tumors in familial adenomatous polyposis: how much do clinical and surgical variables interfere with their development?. Clinics.

[36] Akbulut S., Küçükakçalı Z., Çolak C. (2023). Predicting duodenal cancer risk in patients with familial adenomatous polyposis using machine learning model. Turk J Gastroenterol.

[37] Campos F.G., Sulbaran M., Safatle-Ribeiro A.V., Martinez C.A. (2015). Duodenal adenoma surveillance in patients with familial adenomatous polyposis. World J Gastrointest Endosc.

[38] Campos F.G., Martinez C.A.R., Sulbaran M., Bustamante-Lopez L.A., Safatle-Ribeiro A.V. (2019). Upper gastrointestinal neoplasia in familial adenomatous polyposis: prevalence, endoscopic features and management. J Gastrointest Oncol.

[39] Belchetz L.A., Berk T., Bapat B.V., Cohen Z., Gallinger S. (1996). Changing causes of mortality in patients with familial adenomatous polyposis. Dis Colon Rectum.

[40] Iwama T., Mishima Y., Utsunomiya J. (1993). The impact of familial adenomatous polyposis on the tumorigenesis and mortality at the several organs. Its rational treatment. Ann Surg.

